# Characterization and Evaluation of Composite Biomaterial Bioactive Glass–Polylactic Acid for Bone Tissue Engineering Applications

**DOI:** 10.3390/polym14153034

**Published:** 2022-07-27

**Authors:** Georgina Carbajal-De la Torre, Nancy N. Zurita-Méndez, María de Lourdes Ballesteros-Almanza, Javier Ortiz-Ortiz, Miriam Estévez, Marco A. Espinosa-Medina

**Affiliations:** 1 Facultad de Ingeniería Mecánica, Universidad Michoacana de San Nicolás de Hidalgo, Morelia C.P. 58000, Mexico; nancy.zurita@umich.mx (N.N.Z.-M.); javier.ortiz@umich.mx (J.O.-O.); 2 Facultad de Biología, Universidad Michoacana de San Nicolás de Hidalgo, Morelia C.P. 58000, Mexico; balmanza@umich.mx; 3 Centro de Física Aplicada y Tecnología Avanzada, Universidad Nacional Autónoma de México, Querétaro C.P. 76230, Mexico; miries@fata.unam.mx

**Keywords:** bioactive glass, polylactic acid, scaffolds, electrochemical evaluations

## Abstract

The limitations associated with the clinical use of autographs and allografts are driving efforts to develop relevant and applicable biomaterial substitutes. In this research, 3D porous scaffolds composed of bioactive glass (BG) obtained through the sol-gel technique and polylactic acid (PLA) synthesized via lactic acid (LA) ring-opening polymerization were prepared by the gel-pressing technique. Two different weight compositions were evaluated, namely, BG70-PLA30 and BG30-PLA70. The structure and morphology of the resulting scaffolds were analysed by FTIR, XRD, SEM, and under ASTM F1635 standard characterizations. The results confirmed that BG promotes the formation of a hydroxy-carbonated apatite (HAp) layer on composites when immersed in simulated body fluid (SBF). Biodegradability evaluations were carried out according to the ISO 10993-13:2010 standard. In addition, electrochemical evaluations were performed in both Hank’s and SBF solutions at 37 °C in order to analyse the degradation of the material. This evaluation allowed us to observe that both samples showed an activation mechanism in the early stages followed by pseudo-passivation due to physical bioactive glass characteristics, suggesting an improvement in the formation of the HAp nucleation. The described composites showed excellent resistance to degradation and outstanding suitability for bone tissue engineering applications.

## 1. Introduction

Materials that substitute bone tissues are of great interest to the scientific community, as traumatic injuries and pathologies in which the skeletal structure is damaged are extremely common [1,2,3]. Several years ago, it was thought that human tissues or organs were only replaceable by transplants or metallic and polymeric devices. However, many of these materials can cause an undesirable immune response, leading to inflammation and rejection. Biomaterials based on the SiO_2_–CaO–Na_2_OP_2_O_5_ system, commonly called bioactive glass (BG), have the ability to form bonds with bone and connective tissues; this ability is attributed to the formation of a silica layer with a high surface area and the formation of polycrystalline hydroxyapatite layers on the bioactive glass surface [4,5]. BGs have been studied in soft-tissue engineering applications such as peripheral nerve regeneration and chronic pain treatment as well [6].

BG is frequently obtained by two methods: 1. melt-derived glass, in which the oxides are silica, calcium, phosphate, and sodium precursors, which then undergo further solidification; and 2. sol–gel synthesis, which employs low processing temperatures for an economical method in which the properties can easily be controlled. Overall, the bioglasses obtained by this method exhibited high surface areas and suitable porosity, providing osteogenic potential [7,8,9]. Furthermore, lactic acid (LA) production in its L(+) isomerism is promoted by the intense physical activity of the muscles; although LA is unassimilable by the organism, its produced polymer (polylactic acid, or PLA) has a high biodegradability rate and is bio-compatible, immunologically inert, non-toxic, and absorbable [10]. Consequently, this polymer could be used for the elaboration of a composite biomaterial for bioengineering applications such as controlled drug release systems, bioabsorbable fixation devices, and bone regeneration implants. Through the method of direct polycondensation, it is possible to obtain low molecular weight products, which are important in biomedical applications [11]. As is known, the surface reactions of materials with their biological environment occur a few seconds after they are implanted in the body, interacting with proteins present in the physiological environment; hence, it is important to evaluate the in vitro biological behaviour of the biomaterials. Simulated body fluid (SBF) and Hank’s saline solution at 37 °C are the aqueous media that allow for understanding of the corrosion mechanism of composite biomaterials, as their ionic compositions are close to those of human plasma [8,9,10,11,12].

This research synthesized BG using the sol–gel method, using tetraethyl orthosilicate (TEOS) as an initial precursor. The polymeric material (PLA) was synthesized by the ring-opening polymerization of lactic acid, and they were subsequently mixed by employing a solvent in two different weight compositions (BG70-PLA30 and BG30-PLA70), then deposited by dip coating on 316L stainless steel sheets of about 0.2 mm in thickness. Assessment of the corrosive behaviour in Hank’s solution and simulated body fluid (SBF) was performed using electrochemical techniques. Their bioactivity in PBS was evaluated by the ASTM F1635 standard test method for in vitro degradation testing of poly (L-lactic Acid) resin and fabricated as scaffolds. In contrast, SBF bioactivity was evaluated using the methodology of Kokubo et al. [13]. This project discusses the results of the methods and measurements of the properties of these scaffolds, describes a desirable resistance to degradability and bioactivity in simulated body solutions, and uses extensive electrochemical analysis to evaluate the degradation conditions of the composite biomaterial.

## 2. Materials and Methods

The sol–gel technique was used to synthesize the bioactive glass, while polylactic acid was synthesized via lactic acid (LA) polycondensation. Furthermore, BG, PLA, and BG-PLA composite samples were prepared and characterized by Fourier transform infrared spectroscopy (FTIR), X-Ray diffraction (XRD) analysis, and scanning electron microscopy (SEM). The corrosion behaviour of the composites in Hank’s balanced salt solution and simulated body fluid (SBF) at 37 °C were performed using electrochemical techniques. The biomaterial bioactivity in SBF and phosphate-buffered saline (PBS) was measured each week for 28 days.

### 2.1. PLA Synthesis

In order to reproduce a more efficient PLA production process, the ring-opening polymerization (ROP) method was carried out. The initial lactic acid of reactive grade (Meyer ^®^) was put inside using a rotary evaporator (Hahnshin Scientific Co., model: HS-2000NS, Michoacán, México), applying a 35-rpm rotation, heating temperature, and vacuum at −200 mmHg. Tin(II) 2-ethyl hexanoate (Sigma-Aldrich ^®^ ~95%, Michoacán, México) was added, and the temperature was raised to 175 °C. The reaction takes 6 h under these conditions. The obtained PLA was dissolved in propanone (Meyer ^®^, Michoacán, México) and precipitated with distilled water. The white PLA powder was then washed, filtered, and dried.

### 2.2. BG-PLA Composite Synthesis

All precursors were reactive grade and used without further purification. The method was performed in two steps. In the first step, tetraethyl silicate (98% Sigma-Aldrich ^®^, Michoacán, México) and 0.1 M nitric acid (JT Baker ^®^, Michoacán, México) were mixed at room temperature. Then, triethyl phosphate (TEP: 99.8%, Sigma-Aldrich ^®^, Michoacán, México) and calcium nitrate tetrahydrate (99%, Sigma-Aldrich ^®^, Michoacán, México) were added at intervals. The reaction lasted for an additional hour after the last compound was added. The bioactive glass was obtained in this step. The general synthesis reactions can be observed in Equation (1). In the second step, the material obtained as a gel form was mixed with the PLA obtained in two weight percentages (wt.%), namely, BG30-PLA70 and BG70-PLA30, employing dissolvent propanone (Meyer ^®^, Michoacán, México). The composite biomaterial was first kept in a sealed glass jar at room temperature for ten days and later at 70 °C for three days. Thermal treatment was performed at 120 °C for two days.

(1) $$SiC_{8}H_{20}O_{4}\overset{HNO_{3\;}0.1\;M}{\to }Si{\left(OH\right)}_{4}\overset{TEP,\;Ca{\left(NO_{3}\right)}_{2}.4H_{2}O}{\to }\;{\left(SiO_{2}\right)}_{x}\;{\left(CaO\right)}_{y}{\left(P_{2}O_{5}\right)}_{z}$$

### 2.3. SBF and PBS Preparation

The SBF was prepared according to the Kokubo protocol [13]. The reaction was controlled at a pH of 7.45 ± 0.01. The obtained solution was cooled at 20 °C and kept under refrigeration at 7 °C. At the same time, the PBS solution was obtained by dissolving a phosphate-buffered saline tablet (Sigma^®^, Michoacán, México) in 200 mL of deionized water to obtain a 0.01 M phosphate buffer with 0.0027 M KCl and 0.137 M NaCl contents, with a pH of 7.4 at 25 °C.

### 2.4. BG-PLA Coatings

A 316L stainless steel sheet approximately 0.2 mm thick was used as the substrate material. These sheets were polished using sandpapers of 230, 300, 500, 600, and 1000 grades. The 316L substrate surface was chemically treated by immersion in NaOH (Sigma-Aldrich ^®^, Michoacán, México) 6M solution for 24 h and cleaned with deionized water and acetone. The composite material for the coating application was obtained by dissolving 5 g of BG-PLA powders in 20 mL of acetone (reagent grade, Meyer ^®^, Michoacán, México). The coatings were applied by immersion using the dip-coating method with a 176 mm/min speed rate and residence time of 30 s. The obtained layers were dried at 120 °C for 24 h.

### 2.5. BG-PLA Scaffolds Design

BG-PLA scaffolds were obtained by the gel-pressing technique, in which 10 g of each composite material (BG70-PLA30 and BG30-PLA70) was dissolved in 40 mL of chloroform (CHCl_3_, Meyer ^®^ 99.8%, Michoacán, México). Subsequently, porosity was achieved by particle leaching using NaCl crystals a maximum of 500 µm of diameter in a proportion of 60 wt.% of the weight of the total components. The homogeneous phase was pressed into containers with 0.635 cm diameter and 1.2 cm height. The solvent was first evaporated at room temperature for two days, then heated in an oven at 50 °C for 24 h. The scaffold samples were immersed in distilled water to eliminate salt, and the porosity formation was dried again in an oven and stored in sterile Petri dishes.

### 2.6. BG-PLA Bioactivity in SBF

The main characteristic of bioactivity in SBF is the formation of hydroxyapatite (HAp) on the material surface. For this evaluation, the BG-PLA scaffolds were immersed in triplicate in a polyethylene bottle (three scaffolds per bottle) with SBF solution in a 100 mL/g ratio at a controlled temperature of 37 °C and pH 7.4. Bioactivity measurements were obtained at 7, 14, 21, and 28 days. The nomenclature identification for the samples is shown in Table 1. At the end of each test, the scaffolds were removed from the SBF solution, gently rinsed with deionized water, and allowed to dry for 4 to 5 days in an incubator at 37 °C. The pH of the solution was monitored, and the SBF solution was replaced every week due to the cation concentration decreasing during the experiments. Bioactivity results were complemented by XRD, FTIR, and SEM characterization.

### 2.7. BG-PLA Degradation in PBS

Degradation monitoring was carried out by measuring the change in weight during the sample’s immersion in PBS. Similarly, the BG-PLA pieces were immersed at 37 °C for 1, 2, 3, and 4 weeks, with the weight change measured after each period. The PBS solutions were replaced every seven days. This study was performed according to the standard ISO of 10993-13:2010 [14]. The percentage of weight loss was calculated from Equation (1). The nomenclature identification for the samples is shown in Table 2.

(2) $$\mathrm{Weightloss}\;\left(\%\right)=100\left(\frac{W_{1}{-W}_{2}}{W_{1}}\right)$$

where *W*_1_ and *W*_2_ are the weight of the dry composite before and after immersion, respectively.

### 2.8. FTIR and XRD Characterizations

Fourier transform infrared spectroscopy analysis was performed with a Bruker spectrometer model Tensor 27. The applied measurement range was 4000 to 400 cm^−1^, with a 4 cm^−1^ resolution and sample and background scan times of 32 scans. The samples were obtained by mixing 0.0020 g of the powders and 0.20 g of KBr, then compressed by applying 9.9 tons of pressure for 1 min with a PIKE Technologies CrushIR hydraulic press machine. Then, the compacted sample was characterized with FTIR equipment. XRD measurements were conducted using a D8 Advanced Da-Vinci equipment X-Ray diffractometer. Scans were taken with a 2θ step size of 0.04° from 20° to 90° and a counting time of 0.3 s using Cu Kα radiation. The phases were identified by matching the observed patterns to the entries in the indexing software.

### 2.9. Electrochemical Tests

Electrochemical tests were performed using a potentiostat/galvanostat Gill-AC (ACM Instruments) controlled by a computer. A three-electrode cell arrangement was used with an Ag/AgCl saturated reference electrode (SSCE-RE), platinum wire as an auxiliary electrode (AE), and the coating samples (WE). Hank’s balanced salt solution (Sigma-Aldrich ^®^, Michoacán, México) modified with sodium bicarbonate (without phenol red, calcium chloride, or magnesium sulphate, sterile-filtered, and suitable for cell culture) and simulated body fluid (SBF) at 37 ± 1 °C was the electrolyte used to emulate human body temperature, which was controlled by an electric heating band.

A polarization potential scan obtained potentiodynamic polarization curves (TF) from −500 mV to +1500 mV vs. open circuit potential (OCP) at a scan rate of 1 mV/s. Corrosion current density values, *i_corr_*, and other parameters were calculated using the Tafel extrapolation method between an extrapolation range of ±100 mV around the OCP. Before running the experiments, a 10 min delay time was set until the OCP reached the steady-state condition. The LPR measurements were obtained in a range of ±15 mV vs. the OCP with a scan rate of 1 mV/s every 15 min for 48 h. Polarization resistance (*R_p_*) and current density kinetics were obtained by Ohm’s law and the Stern and Geary equations [15]. The electrochemical impedance spectroscopy (EIS) measurements were carried out at OCP using a voltage signal with an amplitude of 30 mV and a frequency interval between 23,000 and 0.01 Hz.

## 3. Results

### 3.1. X-ray Diffraction Analysis

**Figure 1:**
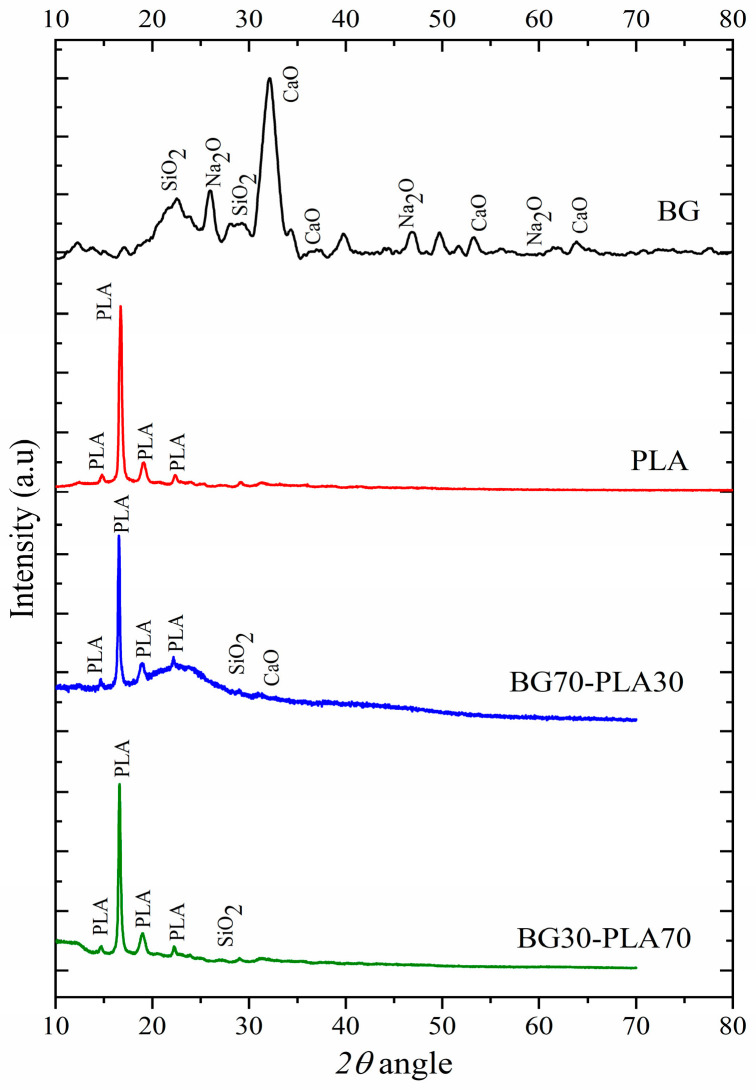
X-ray diffraction for the obtained materials.

The diffraction patterns obtained for the biomaterials are shown in Figure 1. As can be observed, the obtained BG presents a ceramic formulation system composed of SiO_2_–Na_2_O–CaO–P_2_O_5_. The presence of P_2_O_5_ allows formation of a network, promoting the glass crystallization process [16], and induces the formation of a calcium phosphate layer that crystallizes into biomimetic hydroxyapatite due to the incorporation of hydroxide and carbonate ions from the biological fluid [17]. The X-ray diffraction results for the orthorhombic lattice PLA were compared with the crystallographic PDF data 00-064-1624, presenting diffractions in 2θ angles positioned at 12.42°, 16.63°, 19.08°, and 22.3°, correlated to the Miller’s indices of the planes (103), (200), (203), and (211) of the polymeric material. As expected, the composition of the BG-PLA composite at both proportions (BG70-PLA30 and BG30-PLA70) agrees with the presence of each phase.

### 3.2. FTIR Characterization

**Figure 2:**
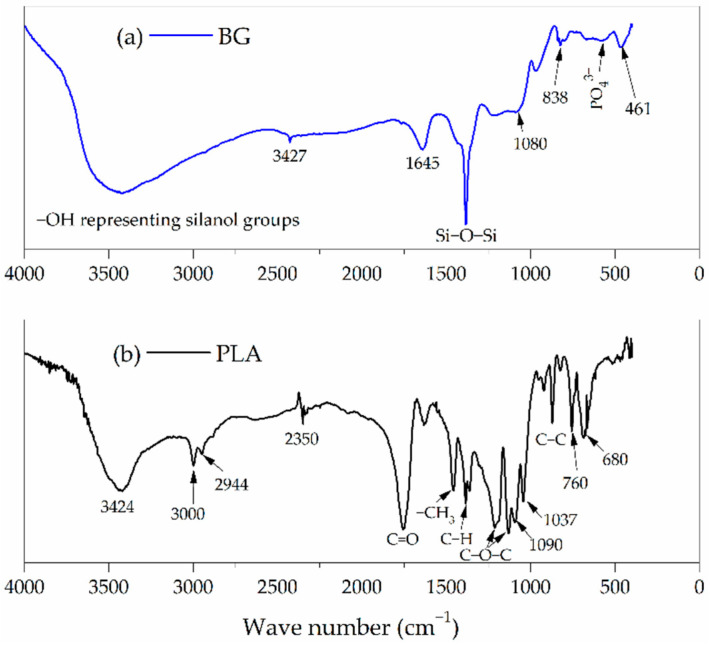
FTIR spectra for the synthesized species (**a**) BG and (**b**) PLA.

Fourier transform infrared spectroscopy with the KBr technique has been used recently to study the structure–composition relationship in various glasses and glass ceramics [18]. The FTIR results for the PLA and BG samples are shown in Figure 2. According to the analysis of the BG spectrum (Figure 2a), absorption peaks could be observed at 1386, 838, and 461 cm^−1^, representing the bending and stretching vibrations of Si−O−Si bonds. The vibrational band with low intensity at 566 cm^−1^ corresponds to the bending vibrations of the phosphate (${\mathrm{PO}}_{4}^{3-}$) groups [19], suggesting that the phosphate can be considered as a network former [20]. The broad band at 3427 cm^−1^ could be ascribed to the vibration of different ${\mathrm{OH}}^{-}$ groups, and represents the surface silanol groups related to different hydroxyl groups. This indicates the superposition of stretching modes of non-hydrogen-bonded silanols (isolated silanol groups) and hydrogen-bonded-silanol (vicinal silanol groups) [21]. As can be observed in the polylactic acid FTIR spectra in Figure 2b, the stretching vibrations of C-C bonds are found at 865 cm^−1^, the asymmetric and symmetric C−O−C stretching peaks are related to 1132 and 1211 cm^−1^, respectively, the C−H symmetric bending can be located at 1375 cm^−1^, −CH_3_ asymmetric bending can be seen at 1457 cm^−1^, C=O stretching bonds are represented at 1755 cm^−1^, and C−H symmetric and asymmetric stretching at the 2944 and 3000 cm^−1^ peaks, respectively [22].

**Figure 3:**
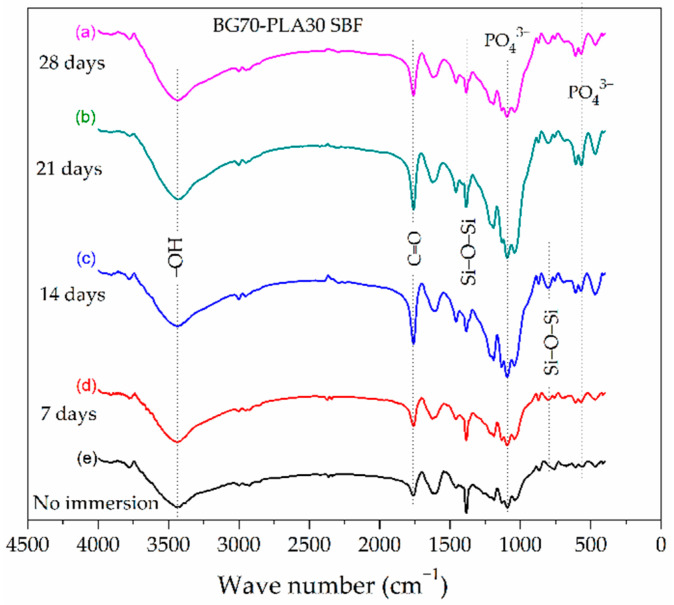
FTIR spectra of the BG70-PLA30 composite after different soaking times in SBF.

The analysis of the bioactivity of the composite scaffolds BG70-PLA30 in SBF by FTIR is shown in Figure 3. The results show vibrational bands related to the silanol groups, C−H, C=O, C−O−C, and P-O bonds; the broad band at 3000–3600 cm^−1^ is present due to the silanol groups on the composite surface. The Hap formation on the surface of the composite immersed in SBF is associated with the presence of the bands around 560–600 cm^−1^, which correspond to the bending vibrations of P−O bonds that are visible in the Si−Na−P system [21]. The FTIR spectrum of the BG70-PLA30 scaffolds shows a broad spectrum, reflecting the Si−O−Si symmetric stretching vibrations.

**Figure 4:**
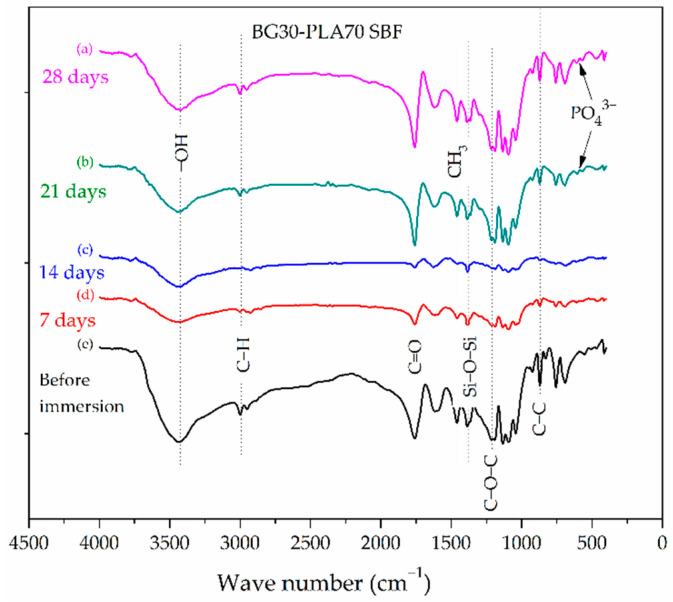
FTIR spectra of the BG30-PLA70 composite after different soaking times in SBF.

The silanol groups at 3500 cm^−1^ are present in the FTIR spectrum of the BG30-PLA70 sample in SBF (Figure 4). Due to the higher composition of PLA in the composite, the presence of stretching vibrations of C-C, C-O-C, and C=O bonds are observed, and are associated with the crystallinity of the PLA phase. The bending and stretching vibrations of Si-O-Si bonds correspond to the BG phase. At 21 and 28 days of immersion, the formation of the Hap phase was observed in the vibrational bands with low intensity at 573 cm^−1^ and 610 cm^−1^, which are related to the bending vibrations of the phosphate (${\mathrm{PO}}_{4}^{3-}$) groups. Chen et al. [23] observed the vibrational bands at 608 and 561 cm^−1^ to be associated with the strengthened intermolecular interaction of the molecules in the crystal lattice in highly-ordered arrangements. Thus, it is possible that the phosphate formation acts as a molecular link. The BG dissolution mechanism in the biological fluids was associated with ions leaching from BG into PBS, followed by decomposition of silica–oxygen bonds of the BG network and redeposition of the calcium and phosphorus ions onto the biomaterial surface [24].

**Figure 5:**
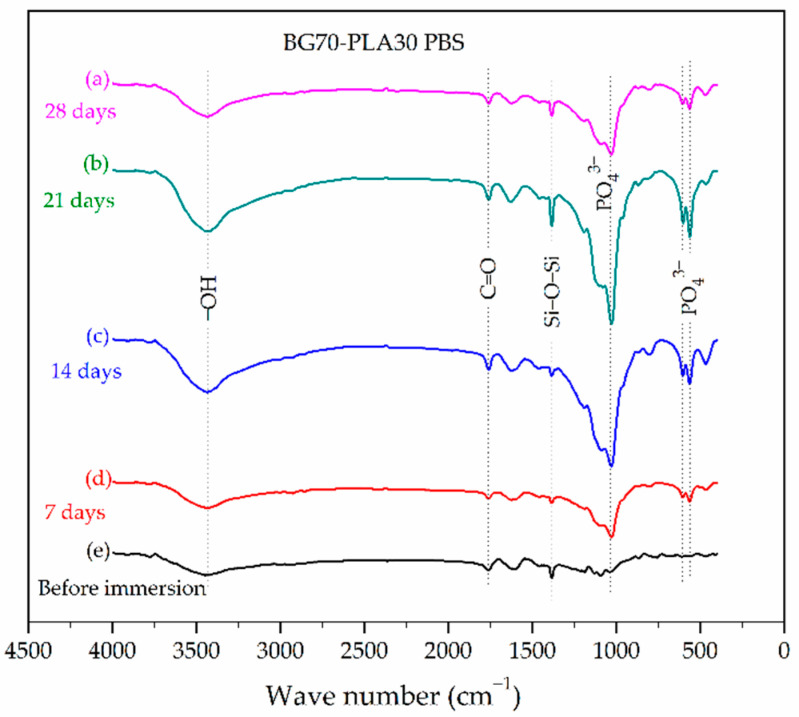
FTIR spectra of the BG70-PLA30 composite after different soaking times in PBS.

**Figure 6:**
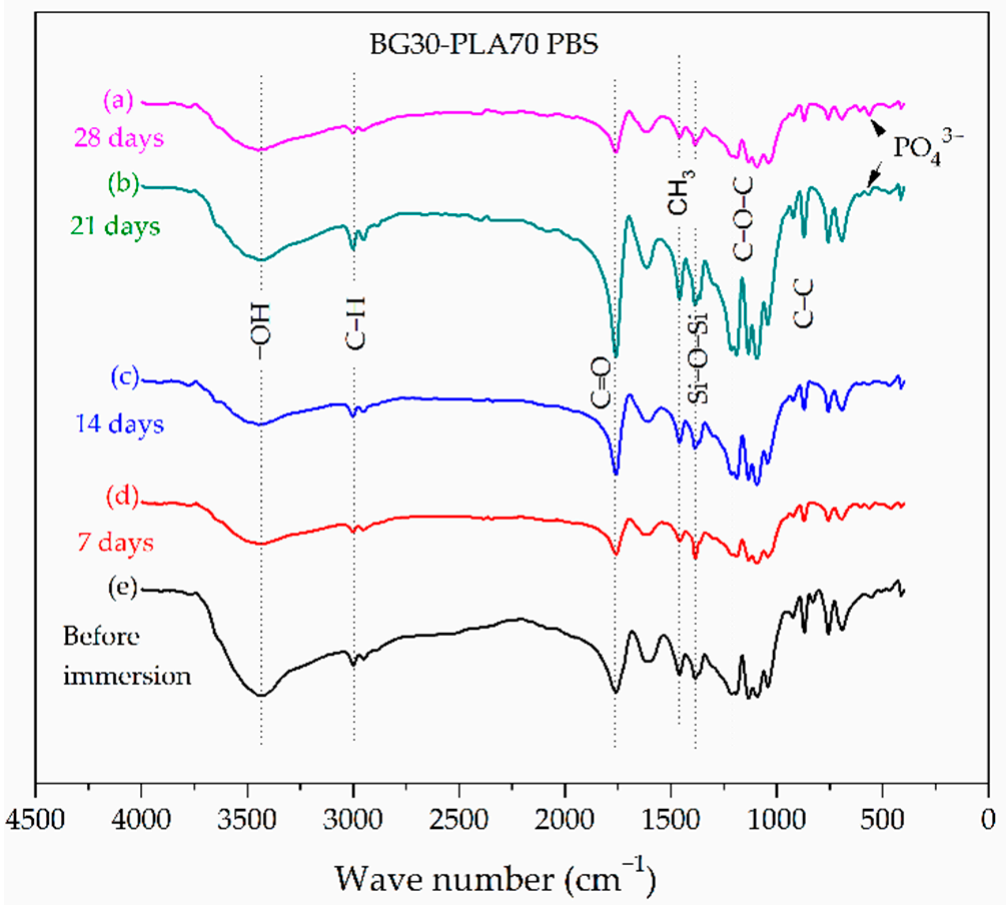
FTIR spectra of the BG30-PLA70 composite after different soaking times in PBS.

Figure 5 and Figure 6 show the FTIR results of the BG-PLA composites in PBS. The FTIR results of the BG70-PLA30 scaffolds in PBS immersed for 7, 14, 21, and 28 days (Figure 5) present consistent degradation due to the presence of the phosphate groups’ bending vibrations, which are more defined with longer immersion times. The mineralization process was associated with the intensity increase of the 1037 cm^−1^ peak due to P-O stretching vibration. The FTIR results for the biomaterial BG30-PLA70 (Figure 6) present the formation of a vibrational band with low intensity at 566 cm^−1^ after 21 days of immersion, corresponding to the deposition of phosphorous ions on the surface. The peak intensity decrease in the vibrational band at 1385 cm^−1^, which corresponds to Si−O−Si bending and stretching vibrations at 28 days of immersion, indicates the process decomposition of the BG phase.

### 3.3. SEM Characterization

**Figure 7:**
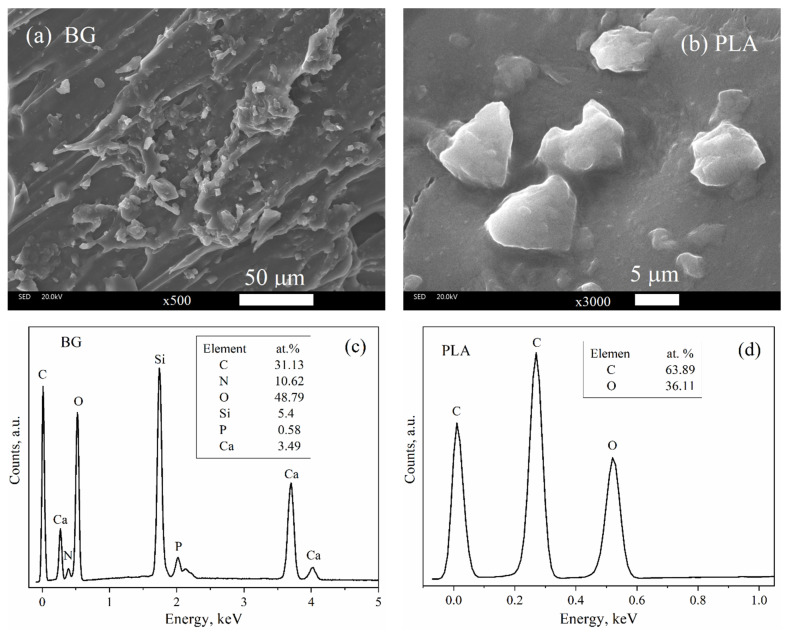
Morphology by SEM of (**a**) BG and (**b**) PLA; EDS chemical analyses of (**c**) BG and (**d**) PLA.

Figure 7a shows that the morphology of the BG presents an irregular morphology and particles with a size between 2.31 to 15.47 μm. Figure 7c shows the chemical composition by EDS of the BG sample, indicating the presence of Ca, O, Si, P, and C, which are constituents of the bioactive bioglass and were observed in the FTIR and XRD characterization results as well. Furthermore, the BG morphology here is similar to that reported by Sharifianjazi et al. [25] and Xia et al. [26]. The PLA morphology is shown in Figure 7b, while its components are indicated in EDS spectrum of Figure 7d.

**Figure 8:**
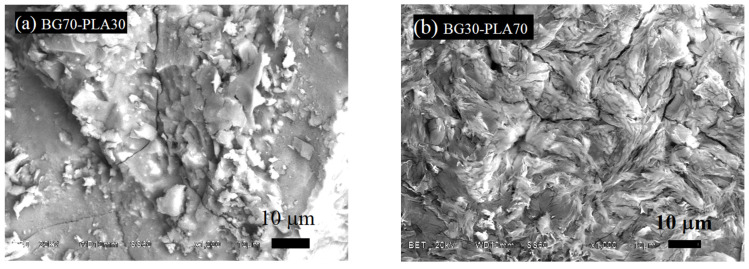
Scaffold SEM analysis for (**a**) BG70-PLA30 and (**b**) BG30-PLA70.

The BG-PLA scaffolds were characterized by SEM as well. The BG70-PLA30 composite (Figure 8a) presented a dense morphology with a homogeneous phase, with no differentiation between the BG and PLA phases. On the other hand, the BG30-PLA70 scaffolds (Figure 8b) showed a cracked surface morphology, which is associated with the presence of tension stress at the grain interfaces of the polymer and BG phase during sintering due to the higher wt.% quantity of the PLA in the composite. The elemental chemical analysis by EDS of both composite samples is shown in Table 3. As expected, the chemical composition for the samples is in agreement with the quantity of the polymeric and vitreous phases, denoting a major percentage of C when the PLA phase was higher in the scaffolds (BG30-PLA70).

### 3.4. Evaluation of BG-PLA Bioactivity in SBF

The evaluation of the bioactivity of the BG-PLA composites was achieved as described in Section 2.3, and is supported by the FTIR (shown in Figure 3 and Figure 4) and SEM characterization. In accordance with Equation (2), the average weight loss of the BG70-PLA30 remained fairly constant over the different time periods of immersion. The average weight loss values ($\bar{X}$) across the four time periods did not show any significant variation, with measured values between 37.85 and 38.5 wt.%, as seen in Table 4. A similar profile was presented by the BG30-PLA70 composite, with an average weight loss of between 39.6 and 38.6 wt.%. Nevertheless, the differences in the weight loss values for both composites during the four time periods are associated with the evolution of HAp formation, which is integrated into the measurement. After immersion, the scaffold composites presented degradation indications due to water interaction in the ion exchange mechanism between the BG/PLA phases and the solution. The water molecules disassociate the Si-O bonds in the BG network forming Si-OH groups, which attracts the ${\mathrm{Ca}}^{2+}$, $H_{2}{\mathrm{PO}}^{4-}$, ${\mathrm{HPO}}_{4}^{2-}$ and ${\mathrm{PO}}_{4}^{3-}$ ions present in the SBF solution. This favourably promoted HAp nucleation site formation on the sample’s surface [23]. The degradation behaviour of the BG (30 wt.%) composite showed an initial increase as a result of water uptake, then a subsequent decrease due to mass loss attributed to the polymer achieving a critical molecular weight sufficiently small to allow diffusion out of the matrix [27], and finally a small mass increase due to room humidity. These three steps are key to sample degradation.

**Figure 9:**
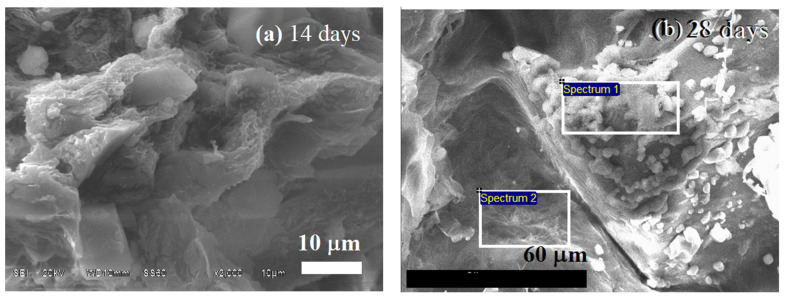
EDS analysis for BG70-PLA30 after (**a**) 14 and (**b**) 28 days of immersion in SBF.

**Figure 10:**
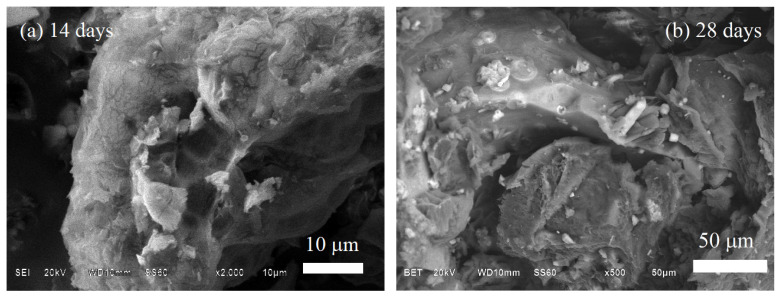
EDS analysis for BG30-PLA70 after (**a**) 14 and (**b**) 28 days of immersion in SBF.

Figure 9 shows the SEM morphology of the BG70-PLA30 scaffolds after 14 days and 28 days of immersion in SBF. The morphology surfaces show the evolution of HAp formation, with greater presence at 28 days of immersion. The EDS chemical composition related to Figure 9 is presented in Table 5 and confirms the HAp growth associated with the quantity of calcium adhesion to the surface (spectrum 2), which is substantial after 28 days of immersion of the biomaterial in SBF. Similarly, Figure 10 shows the SEM morphology of the BG30-PLA70 scaffolds after 14 days and 28 days of immersion in SBF. After 14 days of immersion, the sample morphology shows the presence of cracks on the surface which represent the early stages of degradation, promoting the first interstitial condition for HAp phase nucleation. The EDS results of the chemical analysis of the composite at 14 and 28 days is shown in Table 5. The chemical composition is quite similar in the elements and atomic percentages between both time periods, indicating that the BG concentration in the biomaterial scaffolds is important for optimal bioactivity.

### 3.5. Evaluation of BG-PLA Degradation in PBS

Because biodegradability is an essential property when designing scaffolds, the evaluation of this property was realized and supported by FTIR (Figure 5 and Figure 6) and SEM characterizations. The weight loss of the samples after immersion in phosphate-buffered saline solution (PBS) is shown in Table 4. The degradation behaviour of the BG70-PLA30 sample (blue line) shows the lower percentage change in mass in PBS. This profile can be divided into two regions: an initial increase due to the water uptake from the amorphous areas with the presence of terminal groups, folds, and chains with free rotation, and subsequent mass loss represented by a final decrease related to the degradation rate due to attack on the crystalline areas [28]. Meanwhile, the red line represents the average weight loss profile for the BG30-PLA70 composite in Table 4. The weight loss of the sample was approximately 28% over the four time periods, showing similar behaviour in this solution.

**Figure 11:**
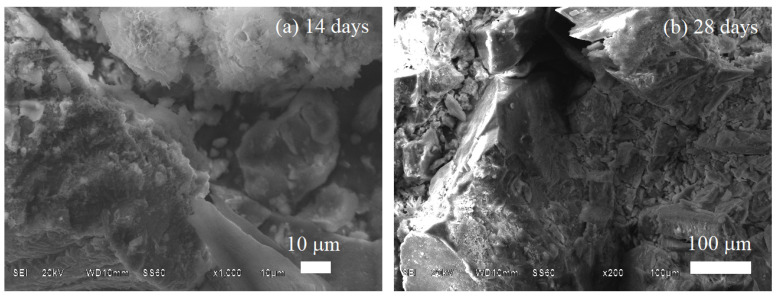
Scaffold SEM analysis for BG70-PLA30 after (**a**) 14 days and (**b**) 28 days of immersion in PBS.

**Figure 12:**
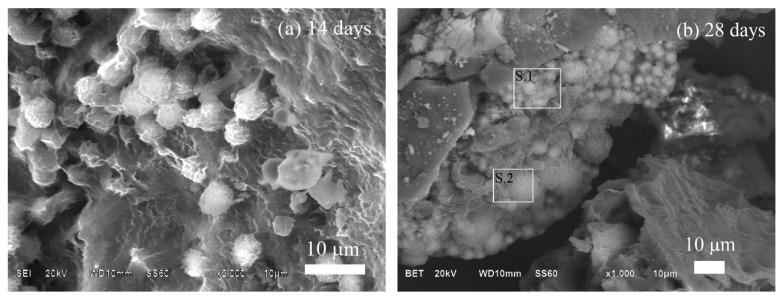
Scaffold SEM analysis for BG30-PLA70 after (**a**) 14 days and (**b**) 28 days of immersion in PBS.

The morphology of the BG70-PLA30 composite after 14 and 28 days immersed in PBS is shown in Figure 11. After 28 days of immersion the surface sample showed more dissolution than at 14 days. The highest mass dissolution occurred at 28 days, diminishing the formation of reaction products deposited on the composite surface (Figure 11b). The sample’s surface did not show higher product growth than the sample immersed for 14 days (Figure 11a). Additionally, the results of EDS analysis in both time periods confirm the degradation behaviour when comparing the elements presented in the samples after 14 and 28 days of soaking in PBS (Table 6). The carbon, sodium, and chloride elements in the initial sample were degraded into the solution after 28 days of immersion, as noted in Table 6. Similarly, the morphology of the degradation of the BG30-PLA70 scaffolds can be observed in Figure 12 after 14 and 28 days of immersion. The formation of spherical growths formed by the HAp phase and the presence of calcium in a high concentration confirms this. For this composite, the results of the EDS chemical analyses shown in Table 5 after 14 and 28 days of immersion present the peak bioactivity of the prepared scaffolds. The increase in Ca content is more evident in the samples with longer immersion times.

### 3.6. Electrochemical Evaluation

This section shows the in vitro results for the biomaterials in Hank’s and SBF solutions; electrochemical techniques were used to identify the mass transport mechanism through the developed biomaterial applied as a coating on the 316L SS substrate.

#### 3.6.1. Potentiodynamic Tests

**Figure 13:**
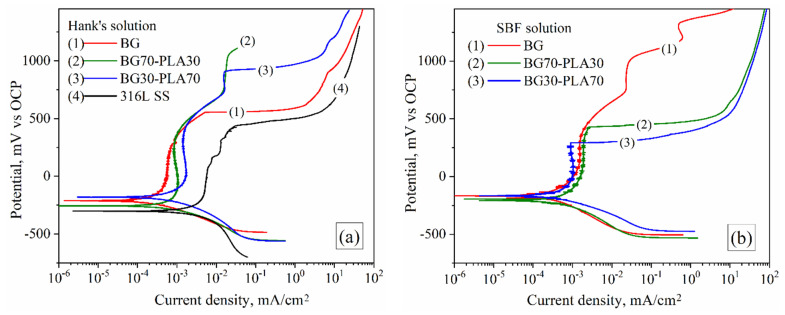
Potentiodynamic behaviour of both composite scaffolds in (**a**) Hank’s saline solution and (**b**) SBF.

Figure 13 shows the corrosion behaviour of the BG-PLA biomaterial samples (BG, BG70-PLA30, and BG30-PLA70) in both Hank’s (Figure 13b) and SBF (Figure 13b) saline solutions at 37 °C. This condition of saline solutions is representative of the behaviour of the biomaterials in corporeal applications. All samples showed an activation mechanism in the early stages, followed by a pseudo-passivation or current limited behaviour associated with the inhibited corrosion due to the physical barrier formed by the coatings. The behaviour presented after activation was associated with the physical bioglass characteristics; as a semiconductor material, charge transfer is limited by this property. After that, a wide over-potential range inhibiting corrosion (as passivation behaviour) up to breakdown over-potential was observed in the coated samples. Table 7 shows the potentiodynamic parameter obtained from polarization plots (Figure 13). In general, *i_corr_* showed low current density values between 0.1 to 0.3 µA/cm^2^, which is lower than the *i_corr_* presented by the 316L SS (around 0.733 µA/cm^2^) under similar conditions (Figure 13a, curve 4). Furthermore, the *i_pass_* values were observed in the same order of magnitude. The coated samples showed a corrosion potential *E_corr_* more positive than the 316L SS as a correlation of minor electrochemical activity, as indicated in Table 7. The electrochemical behaviour of the bioglass materials could be utilized in biomedical applications as biomaterial supports.

#### 3.6.2. LPR Measurements

**Figure 14:**
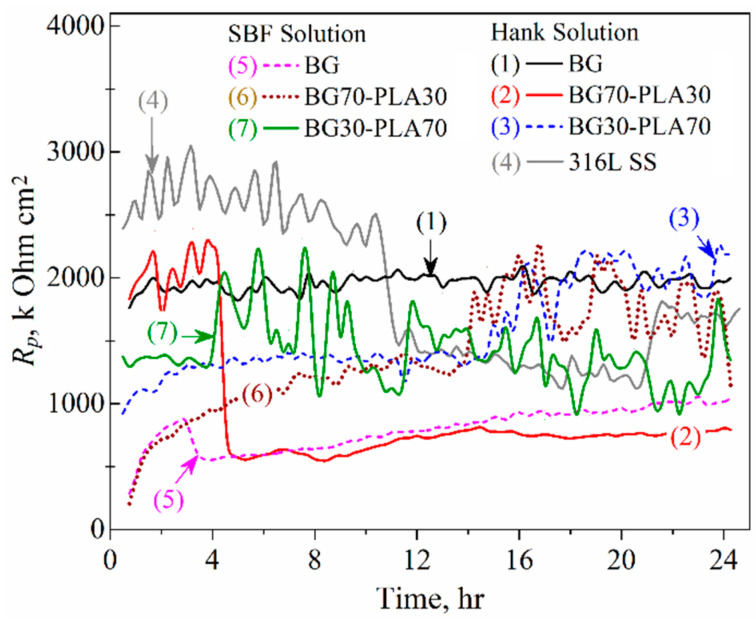
*R_p_* kinetics of the BG-PLA coatings in Hank’s and SBF solutions at 37 °C.

**Figure 15:**
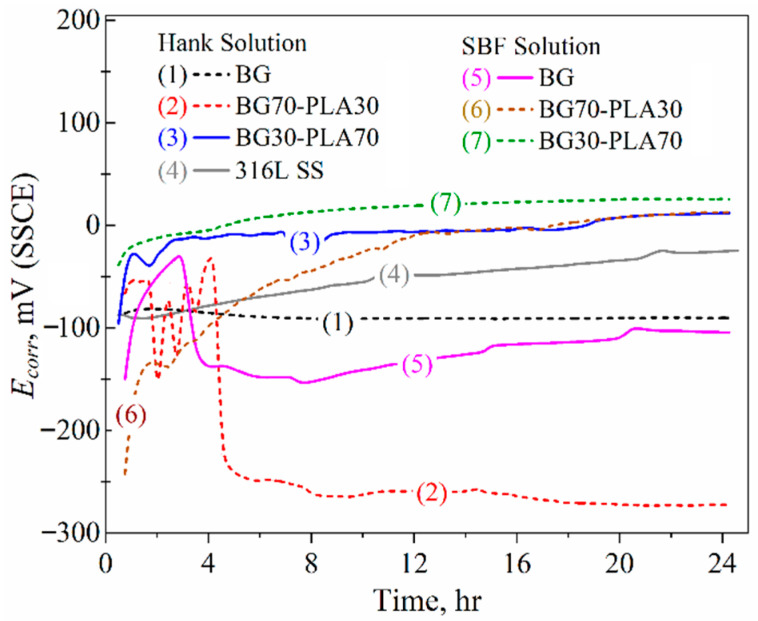
*E_corr_* kinetics of the BG-PLA coatings in Hank’s and SBF solutions at 37 °C.

To observe the behaviour of the coatings as a function of time, linear polarization resistance (LPR) measurements were made. The polarization resistance (*R_p_*) and *E_corr_* kinetics obtained by the LPR measurements of the BG, BG70-PLA30, and BG30-PLA70 coatings in both saline solutions are shown in Figure 14 and Figure 15 (the substrate was measured in Hank’s solution only). In Hank’s solution, the substrate alloy showed the highest *R_p_* values during the first 10 h of immersion (about the 2.5 M Ohm·cm^2^), although it showed a decrease of around 1.2 M Ohm·cm^2^. This was associated with localized anodic dissolution and the breaking of the passive film present from the beginning of immersion due to the activity of chlorine in the solution. However, the BG coating displayed stability during complete immersion, with *R_p_* values around 2 M Ohm·cm^2^ (Figure 14, curve 1), which was associated with the homogeneous and continuous covering of the coating on the substrate. On the other hand, BG coating in the SBF solution showed the lowest *R_p_* kinetic values, between 0.5 to 1 M Ohm·cm^2^.

The addition of PLA to the BG phase caused variability in the Hank’s and SBF solutions In particular, the hybrid coatings in the SBF solution presented increased corrosion resistance in the second half of immersion, as did the BG30-PLA70 in Hank’s solution (Figure 14, curve 3). Although the BG70-PLA30 coating in Hank’s solution did not show *R_p_* kinetics as the others did, the coated samples generally showed improved corrosion resistance in a stable range between 1 to 2 M Ohm·cm^2^. The *R_p_* fluctuations were correlated with the porous characteristics of hybrid coatings that promote a finite diffusion corrosion mechanism, as described below in the EIS results. Likewise, the potential kinetics present the evolution of the activity of the coatings, as can be seen in Figure 15; as a result, the BG70-PLA30 coating in Hank’s solution showed more negative potentials, as did the BG in SBF; thus, the *R_p_* values were the lowest. This behaviour was associated with the characteristics of the coating microstructures. However, the *E_corr_* kinetics of the other coating samples remained stable during the immersion time; the BG30-PLA70 hybrid coating kept the potentials in both solutions higher, in accordance with those shown by the metallic substrate. In addition, the BG70-PLA30 coating in the SBF solution developed exponential growth of the *E_corr_* kinetic to the more positive potentials; thus, the corrosion resistance increased.

**Figure 16:**
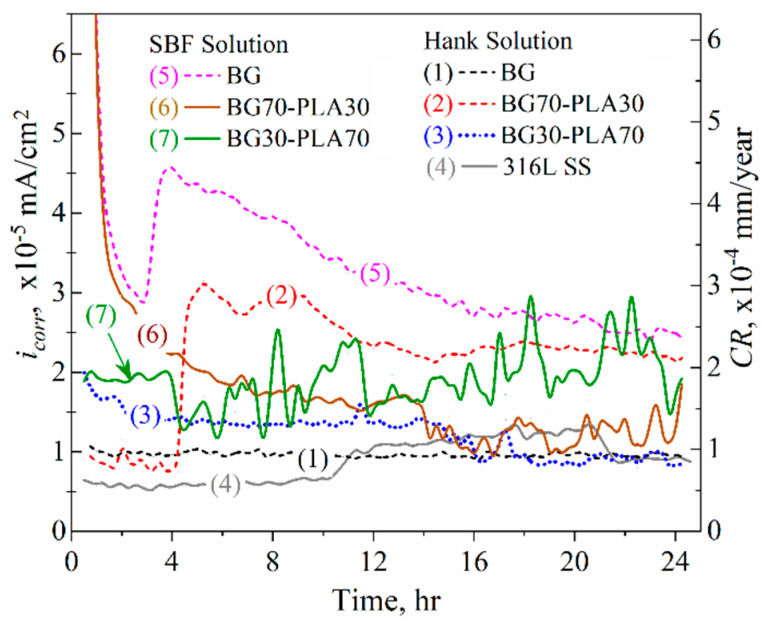
*i_corr_* kinetics for the BG-PLA coatings in Hank’s and SBF solutions at 37 °C.

The kinetic current density (*i_corr_*) showed the opposite behaviour in terms of *R_p_* kinetics because of the indirect correlation of the current density with the resistance, as described by Ohm’s law; these were calculated using the Stern and Geary function [15]. Figure 16 shows the corrosion current behaviour of the coatings in both solutions. According to the *R_p_* results, in Hank’s solution the lowest *i_corr_* values were observed with the BG and the BG30-PLA70 coatings and the 316L SS substrate, as well as the BG30-PLA70 hybrid coating in the SBF solution, with *i_corr_* values around 0.1 µA/cm^2^ in the second half of the immersion time. However, the BG70-PLA30 hybrid coating showed *i_corr_* kinetic instability in the SBF solution. According to the *R_p_* results, the highest current densities were displayed by the BG coating in the SBF solution, followed by the BG70-PLA30 in Hank’s solution (Figure 16 curves 5 and 2, respectively).

The estimation of the corrosion rate (*CR*), as described in the ASTM G102 [15] using the *i_corr_* kinetics (Figure 16 right scale), is valid for the data obtained by the substrate, presenting a *CR* between 0.06 to 0.12 µm/year. These lower *CR* values are a consequence of the chromium oxide protective film formed previously on the self-protected 316L SS alloy. The application of the hybrid coatings did not lead to an increase in corrosion resistance. However, this was not the main purpose of coating the metallic substrate with the BG-PLA biomaterials; rather, it was to improve their functionality due to their high bioactivity, osteoconductivity, and biodegradability for potential applications and physiological functionality as implantable devices. Therefore, the kinetics of the hybrid coatings showed *CR* values as low as the substrate and in the same order of magnitude. In general, the kinetics showed stability of current density and *CR* at around 1 to 2 µm/year during the immersion time, with the exception of the BG coating in SBF solution and the BG70-PLA30 in Hank’s solution.

#### 3.6.3. EIS Analysis

**Figure 17:**
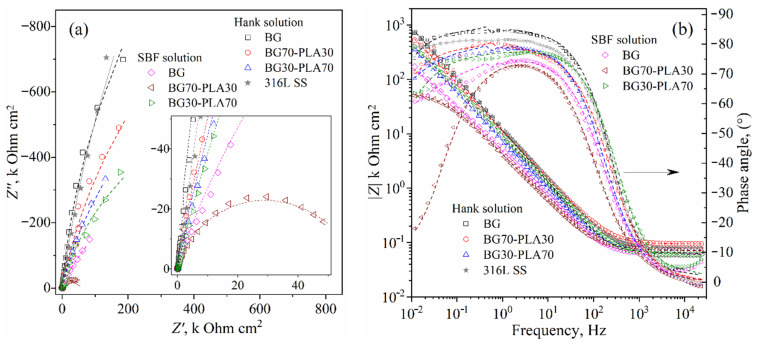
EIS results for BG-PLA coatings obtained at the beginning of immersion in Hank’s and SBF solutions: (**a**) Nyquist and (**b**) Bode plots. Scatter and lines indicate the experimental data and the fitting results, respectively.

**Figure 18:**
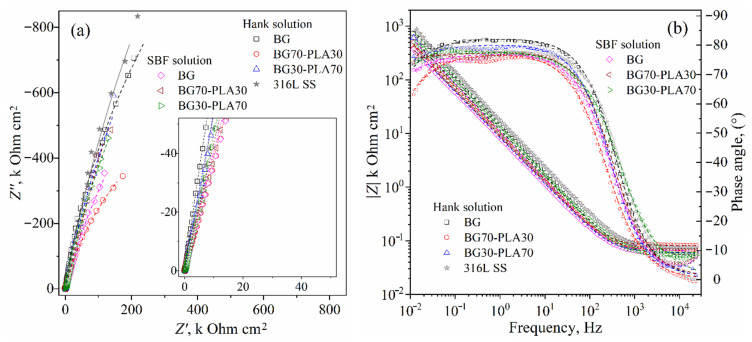
EIS results for BG-PLA coatings obtained after 24 h of immersion in Hank’s and SBF solutions: (**a**) Nyquist and (**b**) Bode plots. Scatter and lines indicate the experimental data and the fitting results, respectively.

Electrochemical impedance spectroscopy (EIS) was used to identify the probable corrosion mechanisms present at the solution/coating and coating/substrate interfaces and through the thickness scale. Additionally, the substrate alloy was evaluated to establish a baseline or reference curve. The 316L SS was evaluated in Hank’s solution, considering that similar results could be obtained in SBF. Two EIS measurements were obtained for the substrate and the coated samples to identify the corrosion mechanisms both at the beginning and after approximately 24 h of immersion. Figure 17 shows the EIS results of the coatings and substrate at the beginning of immersion in the Hank’s and SBF solutions, represented in Nyquist and Bode diagrams (Figure 17a,b, respectively). Likewise, the EIS measurements obtained after 24 h of immersion are presented in Figure 18.

The Nyquist plots obtained at both immersion times (beginning and 24 h.) show the representative form of high resistance corrosion mechanisms for both coated and uncoated samples. The impedance module (|*Z*|) for all materials showed high values above 100 k Ohm·cm^2^, as shown in the Bode plots in Figure 17b and Figure 18b. The substrate as the Nyquist curves of the hybrid coatings presented characteristic capacitive and resistive elements mixed with finite diffusion through a physical barrier composed by the Cr_2_O_3_ film (for uncoated substrate) and Cr_2_O_3_ film/BG-PLA mixed thickness (for the coated samples).

The physical barrier had a high effect on the current density, increasing the time for species diffusion through the scale thickness, retarding the activation mechanism at the metallic interface [29,30]. The phase angle Bode plots show a zone within a wide range of frequencies (from 500 0.5 Hz, approximately) with phase angle values above to 70°, which according to the *R_p_* results described above correspond to the capacitive and resistive behaviour. This could be of interest in biomedical applications as a scaffold in tissue engineering, allowing the controlled transport of mass through the porous microstructure. Similar to the observed LPR results, the BG coating in the SBF solution and the BG70-PLA30 showed lower initial impedance values (Figure 17a). At 24 h of immersion, the total impedance had increased to values within the same order of magnitude as the other coatings, as shown in Figure 18b.

**Figure 19:**
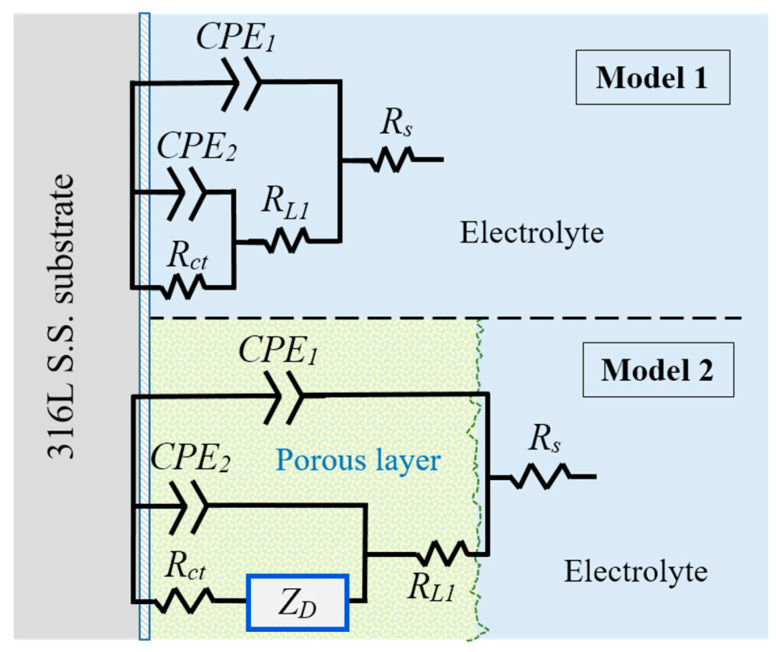
Equivalent circuit models of the corrosion mechanisms observed at the active interfaces: Model 1 for uncoated substrate, Model 2 for BG-PLA coatings.

The proposed corrosion mechanisms were associated with the effect of the microstructural morphology on the coating behaviour composed of the mixed BG and PLA phases at different ratios, as an electrode with a microstructure with a superimposed porous layer [31] acts as a barrier against electron and ion diffusion, reducing the surface area for electrochemical reactions at the metallic interface [29]. Nevertheless, the micro-galvanic cell formation at the coating/substrate could be increased. However, the corrosion behaviour of the substrate is associated with the activation mechanism and with diffusion through the Cr_2_O_3_ protective film. Figure 19 shows the electric circuit models (ECM) that were used as an analogy to better explain the governing corrosion mechanism at the active surfaces and the coating thickness. For the substrate, the analogue ECM correspond to Model 1 (Figure 19), which is composed of the electrolyte resistance (*R_s_*), set up in series with a parallel arrangement of a constant phase element (*CPE*_1_, as capacitive behaviour at the double layer) and the polarization resistance (*R*_*L*1_) of the inner layer (composed of Cr_2_O_3_), which represents the activation mechanism at the Cr_2_O_3_ film/electrolyte interface. The arrangement of *CPE*_2_ in parallel with the *R_ct_* represents the diffusive element presented by the Cr_2_O_3_ protective layer inherent in stainless-steel alloys. When the hybrid coating is applied, the ECM incorporates an element of Warburg diffusion impedance (*Z_D_*)in a serial arrangement before the charge transference resistance (*R_ct_*) at the coating/substrate interface (Model 2 of Figure 19), describing the diffusion through the coating thickness with enough roughness and porosity and allowing the fluid permeation and/or the ion diffusion. Consequently, the electrochemical mechanism governed by limited mass transport was observed.

In the ECMs used here, the *Z_D_* element (finite-length Warburg) represents the short Warburg (*W_s_*) element. In general, the ECM described here represents the analogue equivalent electrical circuit of the impedance for a coated electrode by a hybrid porous layer [31]. The elements of ECM are defined using the following equations:

(3) $$Z\left({CPE}_{i}\right)=\frac{1}{T_{{CPE}_{i}}{\left(j\omega \right)}^{\alpha }}$$

(4) $$Z\left(R_{i}\right){=R}_{S}{,\;R}_{L1}{,\;R}_{ct}$$

(5) $$Z_{W_{s}}=\sigma \frac{\tan h{\left({jT}_{D}\omega \right)}^{P}}{{\left({jT}_{D}\omega \right)}^{P}}$$

where *R_s_*, *R*_*L*1_, and *R_ct_* are the electrolyte resistance, inner layer, and charge transference resistance, respectively, *T_CPEi_* is the *i* constant phase capacitance, and *α* is a dimensionless potential number (0 < α ≤ 1, while α = 1 assumes that *CPE* is a perfect capacitance *C_dl_*). Angular frequency is ω = 2π*f* with *f* = linear frequency, complex number *j* = √(−1), and *Z_f_* is the Faradaic impedance at the metal/scale interface. Hence, the term *T_D_* represents the ratio of scale thickness *L* and the effective diffusion coefficient *D_eff_* of that scale, *T_D_* = *L*^2^ *D_eff_*^−1^ power is between 0 < *P* < 1, and *σ* is the constant of diffusion or the modulus of the Warburg resistance. Here, the *CPE_i_* elements were applied instead of the perfect capacitance for better fitting. Table 8 and Table 9 show the fitting values obtained for each equivalent electric element of the ECM used in the fitting analysis of the experimental data. The lines in Figure 17 and Figure 18 correspond to the fitting results of the experimental data using the proposed ECM.

In the experimental data fitting, the *CPE_i_* elements were applied instead of the perfect capacitance for better fitting. These *CPE* elements were associated with the heterogeneous morphology of the metallic surface and the surface of the coating. For the purposes of EIS analysis, a homogeneously distributed porous microstructure of the BG-PLA coatings was considered, which was formed during the drying and sintering process after applying them to the substrate. Although the coating surface SEM images are not presented here, the fitting Model 2 matches the experimental data associated with a porous microstructure. Additionally, the surface roughness of the substrate and the coatings promotes a depression of the semicircle (Nyquist plot) of the activation process (for the typical *Randles* circuit), and the *α* parameter values (Equation (3)) are lower than unity, as shown in Table 8 and Table 9. Thus, the Nyquist plots present a depression, and the phase angles have lower values (Figure 17 and Figure 18). However, the values of parameter *P* (Equation (5)) were higher than 0.5, which is associated with the Warburg diffusion mechanism and a 45° angle of the phase at low frequencies. Thus, *p* values close to 1 (Table 9) represent a mechanism associated with capacitive behaviour associated with the high resistance characteristic of the coatings. The Bode plots show a combination of the effect of the time delay of the mass transfer mechanism due to the finite diffusion of species through the coating thickness and the resistive characteristic of the Cr_2_O_3_ inner layer, causing a wide range in the loop, with about 70–80° of the phase angle formed from the middle to the low frequencies. Similar results have been previously reported [32], and were associated with high resistance and capacitive behaviour.

## 4. Discussion

The electrochemical results with the porous hybrid coatings allow for mass transport and fluid permeation, which was observed in this work, suggesting potential applications in the tissue engineering area, results which are of interest for future study. Although study of the coatings’ bioactivity for bone regeneration was not within the scope of this work, it is proposed for further study. Accordingly, the formation of particles with Ca and P contents during the bioactivity testing of BG-PLA in SBF and PBS solutions (described in Section 3.4 and Section 3.5 above) due to the interaction of the H_2_O molecules with the Si-O bonds in the BG microstructure promoted the formation of Si−OH groups, which attract the ions of ${\mathrm{Ca}}^{2+}$, $H_{2}{\mathrm{PO}}^{4-}$, ${\mathrm{HPO}}_{4}^{2-}$, and ${\mathrm{PO}}_{4}^{3-}$ in the SBF solution. This favours the HAp nucleation sites, and they precipitate after a period of immersion time; similarly, observation has been made for a hybrid composite with a PCL matrix [29,33,34]. The amorphous inorganic formation of the nuclei crystallizes into the apatite phase [34], and the addition of nanopowders to the polymer matrix can improves apatite nucleation [29]. In accordance with these results, the BG phased improves of the formation of the HAp phase, as has previously been suggested.

Based on the *R_p_* kinetics behaviour, the proposed mechanisms associated with the EIS fitting results, and the formation of HAp particles, the physical characteristics of the microstructure of the coatings allowed redox reactions to take place at the porous surface. Therefore, ${\mathrm{Ca}}^{2+}$, $H_{2}{\mathrm{PO}}^{4-}$ and ${\mathrm{HPO}}_{4}^{2-}$ concentrations were increased at those sites and the associated current density was added to the total measured at the metallic surface. Because the application of the coatings formed a physical barrier at the metallic surface, the charge transfer consequently decreases and the corrosion resistance should increase. However, the presence of intermediate electrochemical reactions through the coating thickness maintains *R_p_* kinetics values of the coated samples slightly lower than those presented by the substrate. Thus, the electrochemical results support the potential application of the BG-PLA composite in biomedical applications. Consequently, further studies to determine the in vitro degradation behaviour and adhesion performance of the hybrid coatings will be undertaken.

## 5. Conclusions

In summary, BG-PLA composite scaffolds with two different compositions synthetized by the sol–gel technique were evaluated and characterised. The morphology of the BG70-PLA30 composite structure was dense, with a well-distributed phase. The surface morphology of the BG30-PLA70 composite presented crack formation associated with tension stress concentrations in the polymeric phase during the drying process. Their potential as bone tissue engineering scaffolds was assessed by in vitro testing using Hank’s and SBF solutions, confirming the bioactivity of the composites by their ability to form HAp on the surfaces and their adequate biodegradation when immersed in PBS after 21 days of immersion. Both properties were confirmed by SEM and FTIR characterization.

The electrochemical evaluation of the scaffolds in Hank’s saline solution and SBF as a coating in a 316L SS substrate allowed us to observe that both samples showed activation mechanisms at the early stages, followed by pseudo-passivation or current-limited behaviour due to the physical characteristics of the bioactive glass, which suggests that improvements in the formation of HAp nucleation consequently allow redox reactions at the surface of the coating.

## Figures and Tables

**Table 1:** Identification of samples evaluated in SBF.

Sample BG70-PLA30	SBF Evaluation (Days)	Sample BG30-PLA70	SBF Evaluation (Days)
BG70-PLA30-SBF-7	7	BG30-PLA70-SBF-7	7
BG70-PLA30-SBF-14	14	BG30-PLA70-SBF-14	14
BG70-PLA30-SBF-21	21	BG30-PLA70-SBF-21	21
BG70-PLA30-SBF-28	28	BG30-PLA70-SBF-28	28

**Table 2:** Identification of samples evaluated in PBS.

Sample BG70-PLA30	PBS Evaluation (Days)	Sample BG30-PLA70	PBS Evaluation (Days)
BG70-PLA30-PBS-7	7	BG30-PLA70-PBS-7	7
BG70-PLA30-PBS-14	14	BG30-PLA70-PBS-14	14
BG70-PLA30-PBS-21	21	BG30-PLA70-PBS-21	21
BG70-PLA30-PBS-28	28	BG30-PLA70-PBS-28	28

**Table 3:** Energy-dispersive X-ray spectroscopy for both compositions.

EDS	BG70-PLA30	BG30-PLA70
Element	at.%	at.%
C	38.485	50.103
O	48.356	46.811
Si	9.704	0.588
Ca	3.454	2.498

**Table 4:** Average weight loss ($\bar{X}$) and standard deviation (***S_X_***) for the scaffolds immersed in SBF and PBS over time periods of 7, 14, 21, and 28 days.

Time, (Days)	SBF				PBS			
	BG30 PLA70		BG70 PLA30		BG30 PLA70		BG70 PLA30	
	$\bar{x},\;(\%)$	*S_X_*	$\bar{x},\;(\%)$	*S_X_*	$\bar{x},\;(\%)$	*S_X_*	$\bar{x},\;(\%)$	*S_X_*
7	39.60	1.34	37.86	1.15	27.80	1.86	24.12	0.03
14	38.88	0.81	38.95	0.27	28.42	1.32	22.42	0.22
21	39.72	1.47	39.33	0.04	28.05	1.06	23.00	0.57
28	38.60	0.92	38.53	0.23	27.91	0.32	24.93	1.06

**Table 5:** EDS chemical analysis for BG-PLA composites after immersion in SBF.

Element at.%	BG70-PLA30			BG30-PLA70	
	14 Days	28 Days		14 Days	28 Days
		S. 1	S. 2		
C	41.25			58.97	51.34
O	45.59	37.9	58.7	34.58	43.6
Na	0.88	25.1	17.3	2.35	1.26
Si	4.82	13.5	8.7	1.05	2.59
P	2.91			0.24	
Cl	0.41	19.1	9.15	2.02	0.85
Ca	4.13	4.41	6.15	0.8	0.36

**Table 6:** EDS chemical analysis for BG-PLA composites after immersion in PBS.

Element at.%	BG70-PLA30		BG30-PLA70		
	14 Days	28 Days	14 Days	28 Days	
				S.1	S.2
C	18.96		35.73	34.87	16.31
O	62.28	72.89	51.23	44.62	57.6
Na	1.07		0.85	1.44	0.38
Si	7.28	22.54	4.82	0.89	1.42
P	0.7	1.37	3.08	6.85	3.7
Cl	0.82		0.39		
Ca	8.88	3.2	3.9	11.33	20.6

**Table 7:** Comparison of potential of corrosion between the samples evaluated in Hank’s and SBF solutions.

Solution	Sample	*i_corr_*	*E_corr_*	*β_a_*	*β_c_*	*E_transp_*	*i_pass_*
		µA/cm^2^	mV	mV	mV	mV	µA/cm^2^
Hank	BG	0.108	−211.7	123	67	545	0.593
	BG70-PLA30	0.241	−257.5	111	62	394	0.817
	BG30-PLA70	0.236	−181.1	92	79	448	1.540
	316L SS	0.733	−301.8	75	67	396	6.310
SBF	BG	0.145	−166.9	134	103	440	1.490
	BG70-PLA30	0.228	−210.0	135	76	428	0.149
	BG30-PLA70	0.190	−168.0	117	122	289	1.040

**Table 8:** EIS Parameters obtained by substrate experimental data fitting (Model 1, Figure 19).

316L SS	Time Immersion	
Model 1	Beginning	24 h
*R_s_* (Ω cm^2^)	78	75
*T*_*CPE*1_ (µF cm^2^)	11.88	10.28
*α*_1_ (*)	0.924	0.896
*R*_*L*1_ (Ω cm^2^)	167	80,654
*T*_*CPE*1_ (µF cm^2^)	2.911	1.032
*α*_2_ (*)	0.892	0.875
*R_ct_* (k Ω cm^2^)	8250.5	8.693

**Table 9:** EIS Parameters obtained by fitting the experimental data for the BG-PLA coatings using Model 2, Figure 19.

**Model 2**	**Hank’s Solution**			**SBF Solution**		
	**Measurement at the Beginning of Immersion**					
	**BG**	**BG70-PLA30**	**BG30-PLA70**	**BG**	**BG70-PLA30**	**BG30P-LA70**
*R_s_* (Ω cm^2^)	62	94	71	61	71	57
*T*_*CPE*1_ (µF cm^2^)	2.316	7.215	1.693	8.954	3.376	15.82
*α*_1_ (*)	0.982	0.884	0.415	0.447	0.867	0.811
*R*_*L*1_ (Ω cm^2^)	6.41	1.88	1.24	0.70	52.95	12.52
*T*_*CPE*1_ (µF cm^2^)	12.77	10.23	25.07	38.09	15.52	6.073
*α*_2_ (*)	0.939	0.883	0.896	0.867	0.822	0.969
*σ* (k Ω cm^2^/s)	4842.1	3122.1	4561.9	2847.2	17.76	1025.9
*T_D_* (s)	1.019	0.872	0.967	0.982	7.8 × 10^−16^	1.7 × 10^−16^
*P* (*)	0.942	0.935	0.971	0.976	0.937	0.941
*R_ct_* (k Ω cm^2^)	15.84	22.76	5.07	3.77	40.35	476.26
	**Measurement at the 24 h**					
	**BG**	**BG70-PLA30**	**BG30-PLA70**	**BG**	**BG70-PLA30**	**BG30-PLA70**
*R_s_* (Ω cm^2^)	60	81	64	58	63	53
*T*_*CPE*1_ (µF cm^2^)	10.80	22.34	15.80	19.18	17.49	8.05
*α*_1_ (*)	0.900	0.848	0.874	0.833	0.840	0.880
*R*_*L*1_ (Ω cm^2^)	12.94	3.08	20.36	14.83	20.88	20.63
*T*_*CPE*1_ (µF cm^2^)	2.285	1.426	2.456	5.729	2.471	8.751
*α*_2_ (*)	0.979	0.992	0.992	0.934	0.992	0.852
*σ* (k Ω cm^2^/s)	2070.7	825.02	5595.5	3390.4	10,397	6207.1
*T_D_* (s)	2.5 × 10^−16^	3.3 × 10^−11^	1.9 × 10^−17^	5.6 × 10^−18^	6.5 × 10^−16^	6.17
*P* (*)	0.811	0.830	0.754	0.772	0.581	0.847
*R_ct_* (k Ω cm^2^)	2612.7	828.52	653.23	83.42	10,252	5.07

Note: (*) Dimensionless Warburg element.

## Data Availability

The datasets generated during the current study are available from the corresponding author on reasonable request.
